# Mobile and cordless telephones, serum transthyretin and the blood-cerebrospinal fluid barrier: a cross-sectional study

**DOI:** 10.1186/1476-069X-8-19

**Published:** 2009-04-21

**Authors:** Fredrik Söderqvist, Michael Carlberg, Lennart Hardell

**Affiliations:** 1 Department of Oncology, University Hospital, SE-701 85 Örebro, Sweden; 2 School of Health and Medical Sciences, University of Örebro, SE-701 82 Örebro, Sweden

## Abstract

**Background:**

Whether low-intensity radiofrequency radiation damages the blood-brain barrier has long been debated, but little or no consideration has been given to the blood-cerebrospinal fluid barrier. In this cross-sectional study we tested whether long-term and/or short-term use of wireless telephones was associated with changes in the serum transthyretin level, indicating altered transthyretin concentration in the cerebrospinal fluid, possibly reflecting an effect of radiation.

**Methods:**

One thousand subjects, 500 of each sex aged 18–65 years, were randomly recruited using the population registry. Data on wireless telephone use were assessed by a postal questionnaire and blood samples were analyzed for serum transthyretin concentrations determined by standard immunonephelometric techniques on a BN Prospec^® ^instrument.

**Results:**

The response rate was 31.4%. Logistic regression of dichotomized TTR serum levels with a cut-point of 0.31 g/l on wireless telephone use yielded increased odds ratios that were statistically not significant. Linear regression of time since first use overall and on the day that blood was withdrawn gave different results for males and females: for men significantly higher serum concentrations of TTR were seen the longer an analogue telephone or a mobile and cordless desktop telephone combined had been used, and in contrast, significantly lower serum levels were seen the longer an UMTS telephone had been used. Adjustment for fractions of use of the different telephone types did not modify the effect for cumulative use or years since first use for mobile telephone and DECT, combined. For women, linear regression gave a significant association for short-term use of mobile and cordless telephones combined, indicating that the sooner blood was withdrawn after the most recent telephone call, the higher the expected transthyretin concentration.

**Conclusion:**

In this hypothesis-generating descriptive study time since first use of mobile telephones and DECT combined was significantly associated with higher TTR levels regardless of how much each telephone type had been used. Regarding short-term use, significantly higher TTR concentrations were seen in women the sooner blood was withdrawn after the most recent telephone call on that day.

## Background

The use of wireless telephones has reached one hundred percent prevalence in many countries. Studies on the potentially harmful biological effects of the radiofrequency fields (RF) emanating from these devices are thus pertinent. Dysfunction of the blood-brain barrier (BBB) is one such effect that has long been debated (for reviews, see Nittby et al. [1] and Orendacova et al. [2]). A barrier that has received much less attention, though it also serves to maintain brain homeostasis by separating the central nervous system from the blood stream, is the so-called blood-cerebrospinal fluid barrier (BCSFB). These brain barriers should be given equal importance as both are implicated in chronic neurodegenerative brain diseases [3]. While the BBB separates or controls the traffic of molecules from the blood to the cerebral interstitial fluid, the BCSFB is a discontinuity in the circulation between the blood and cerebrospinal fluid (CSF). The former is made of endothelial cells and the latter of epithelial cells. A leakage or an alteration in the turnover of brain-derived proteins may be used to evaluate the integrity of these barriers, possibly reflecting an effect of RF.

We previously reported on a possible association between serum concentrations of the calcium-binding protein S100B and self-reported use of wireless telephones among healthy Swedish adults [4]. Mainly synthesized by the end-feet of astrocytes, S100B has been described as a suitable marker of integrity of the BBB [5-8]. In the present paper, using the same data, we report the results of analysis of serum levels of transthyretin (TTR), a key CSF protein. TTR could potentially serve as marker of BCSFB dysfunction.

TTR, also known as prealbumin, is a plasma and CSF carrier of thyroxin and retinol and is also described as sequestering amyloid beta peptide in the brain [9]. Its major sites of synthesis are the liver, the choroid plexus (CP) and the retinal pigment epithelium. TTR is used in clinical practice as a marker in several conditions, such as predicting outcome for critically ill patients [10], in Alzheimer's disease [11,12], amyloidosis [13], inflammation and malnutrition [14].

With regard to synthesis in the brain, TTR, unlike S100B, is not expressed by perivascular astrocytes [15]. It is mainly produced by the epithelial cells of the CP located in the four ventricles, representing about 25% of the CSF protein [16]. CP expands to fill nearly all the cerebral ventricles and has brush-type borders, microvilli, on the apical side. Once filtered by these brushes of microvilli, the CSF flows from the lateral ventricles through the third and fourth ventricles to the subarachnoid space. From there the fluid spreads over the entire surfaces of the brain and spinal cord.

Most of the CSF is emptied into the blood in the venous sinuses by way of arachnoid granulations. These protrusions are particularly abundant in the superior sagital sinuses. The passage of CSF into the venous sinuses is caused by hydrostatic pressure, which is higher in the subarachnoid space, about 15 cm H_2_O, than in the sinuses, 7–8 cm H_2_O [17]. Concentrations of TTR in healthy adults have been reported to be in the range 0.017–0.025 g/L in the CSF [18-20] and 0.20–0.40 g/L in the plasma [21].

Several features make the BCSFB and TTR secretion by the CP particularly interesting for studying a possible effect of RF. Firstly, owing to the enlarged area of microvilli, the CP has a total surface within the same order of magnitude as the BBB [22]. Secondly, it has a rapid blood supply, about 10-fold faster than other regions of the brain [23], with extremely fenestrated endothelial cells that are also quite leaky [24]. Thus, large molecules can readily pass into the connective tissue, but are prevented from further entering the CSF by the tight junctions between epithelial cells. Thirdly, however, these junctions in the BCSFB are leakier than those between the endothelial cells of the BBB [25,26], which would increase the likelihood of insult to the choroidal tissue by toxic materials originating from the blood. Finally, the CP, especially in the lateral ventricles, may be associated with the effects of neurotoxic agents owing to its anatomical location close to several brain structures, among them the hippocampus.

In the present report we tested whether long-term and/or short-term use of wireless telephones was associated with changed concentrations of TTR in the serum as a marker of alterations in CSF TTR, possibly reflecting an effect of RF.

## Methods

### Data collection

One thousand subjects, 500 of each sex, aged 18–65 years and living in the municipality of Örebro in Sweden, were randomly recruited using the population registry. They received a letter of information and were asked to give informed consent to leave blood and have it stored in a so-called Biobank. Respondents who chose to take part then received further information about the purpose of the study. They were asked to complete a postal questionnaire that covered different topics such as background characteristics including employment history, use of wireless telephones (mobile telephones (analogue NMT, digital GSM, UMTS) and DECT), various other exposures such as X-rays, chemicals or radiation therapy and finally health- and lifestyle-related questions such as physical exercise and diseases. The first invitations were sent out in March 2007 and data collection was completed by the end of November 2007. A more detailed description of the study design was presented in the publication about S100B based on the same data [4]. The study was approved by the regional ethical vetting board of Uppsala University.

### Analytical methods

Participants were asked if possible to leave blood samples at Örebro University Hospital in the afternoon at the end of a working week. All samples were taken by a nurse at the chemical laboratory after which they were centrifuged and the supernatant decanted and frozen immediately. Serum TTR concentrations were determined by standard immunonephelometric techniques using commercially-available reagents from Dade Behring GmbH (Marburg, Germany) on a BN Prospec^® ^instrument (Siemens Healthcare Diagnostics). The total coefficients of variation were 3.4% and 5.5% at concentrations of 0.13 and 0.28 g/L respectively. All results are expressed in g/L.

### Statistical Analysis

Frequency tables were produced and explanatory factors were analyzed by the Wilcoxon rank-sum test for one-way comparisons. In all tests the significance level was set at 0.05. Following log-transformation of TTR values to normalize the distribution, linear regression was used to test an association between long- or short-term trends in wireless telephone use and serum concentrations of TTR. Standardized β coefficients are used throughout.

Short-term use of wireless telephones and serum concentrations of TTR were examined by comparing use on the day the blood sample was left; long-term use by comparing cumulative use and years since start of use, as well as fractions of total duration for the different telephone types. To avoid multicollinearity, the fraction of digital phone, which was the most frequently used, was omitted in the linear analysis of long-term use.

The Kruskal-Wallis test was used to analyze the frequencies of use in calls per day and the Wilcoxon rank-sum test to analyze usage in minutes (min) per day. Unconditional logistic regression was used to calculate odds ratios (OR) and 95% confidence intervals (CI) for exposed vs. unexposed in relation to elevated serum levels of TTR. As far as we are aware there is no established cut-off for determining 'normal' and 'elevated' levels of TTR in current use in clinical practice. We therefore chose the third quartile as cut-off (0.31 g/L serum) so that a sufficient number (n) of subjects would be included in the group with 'elevated' concentrations. Because of the low number of users who were literally unexposed (n = 3), definitions of exposed and unexposed in the logistic regression differed depending on the endpoint of interest. The 'unexposed' in the analysis of serum TTR and short-term use were those who reported not having used any type of wireless telephone on the day the blood was drawn; the 'unexposed' in the analyses of long-term use were those with non-use and the quartile with the lowest overall use of mobile telephones and DECT (≤446 hours). All analyses were done using StataSE 10.1 (Stata/SE 10.1 for Windows; StataCorp, College Station Texas, USA).

## Results

### Descriptive analysis

One thousand persons were invited to take part in the study and 314 participated by answering the questionnaire and giving blood. TTR data were missing for one subject so the final analysis included 313 persons. Descriptive analysis showed that women were more likely than men to participate (probability (p) = 0.001; χ^2^-test) and that the mean age was higher among participants than non-participants (p = 0.0001; t-test) (Table 1).

**Table 1:** Descriptive data for the study

	Men	Women	Total
Total included	500	500	1 000

-Age: mean, median	41.8, 41.0	41.6, 42.0	41.7, 42.0
Range	18–65	18–65	18–65

Declined to participate	127	156	283

Did not answer inquiry	196	116	312

Answered yes to participation	177	228	405

-Did not give blood	44	44	88

Participated	133	184	317

-Did not answer the questionnaire	0	2	2

-Unknown date for drawing blood	0	1	1

-Missing data, transthyretin	0	1	1

Final participants	133	180	313

-Age: mean, median	47.1, 50.0	44.4, 46.0	45.6, 47.0
Range	18–65	18–64	18–65

Age and gender predicted TTR levels whereas time, day or month of drawing blood did not (Table 2). Men had higher concentrations than women and participants >47 years age had higher serum levels than younger ones, though when stratification was done on gender a significant difference only remained for women (p = 0.02; p = 0.72 for men). Among the explanatory factors assessed by the self-administered questionnaire hypertension, body mass index (BMI), smoking and use of oral snuff were all statistically significantly related to TTR concentrations, though they had little or no effect when adjusted for in the analyses. Use of detergents was of borderline significance (p = 0.049) in women but was based on only 5 subjects, so no adjustment was made for this variable. Occupation, workload, head or neck X-ray, external radiation, physical activity, and various disease conditions did not predict serum TTR. In further linear and regression analysis, age was adjusted for in all analyses while adjustment for sex was applied only in analyses of total samples.

**Table 2:** Descriptive data for serum transthyretin (g/L)

	n	Mean	Median	Min	Max	p
Transthyretin, total	313	0.277	0.270	0.170	0.480	-

Age (cut-point at median)						

≤ 47 years	158	0.271	0.260	0.170	0.480	0.02

> 47 years	155	0.284	0.280	0.180	0.470	

Gender						

Men	133	0.307	0.300	0.180	0.480	<0.0001

Women	180	0.256	0.250	0.170	0.400	

Time (hour) when blood was drawn						

06:00–12:00	65	0.286	0.270	0.170	0.420	0.12

12:01–16:00	248	0.275	0.270	0.170	0.480	

Day when blood was drawn						

Mon-Thur	174	0.276	0.260	0.170	0.440	0.77

Fri-Sun	139	0.279	0.270	0.170	0.480	

Month when blood was drawn						

March-June	228	0.278	0.270	0.170	0.480	0.94

July-October	85	0.277	0.270	0.170	0.400	

The Wilcoxon rank-sum was used to calculate p-values.

### Serum transthyretin levels and use of wireless telephones

Logistic regression analysis of serum TTR and long-term total use of wireless telephones yielded OR = 1.2, CI = 0.6 – 2.4 (Table 3). Analysis of years since first use of overall use of wireless telephones for periods of more than 5 and 10 years both yielded statistically insignificant results; OR = 1.2, CI = 0.6–2.5 and OR = 1.5, CI = 0.7–3.1, respectively. For mobile telephones the highest OR was found among those who reported > 10 years since first use, whereas OR for DECT did not change with time since first use. Also, higher ORs were found for men than for women.

**Table 3:** Logistic regression analysis for serum transthyretin and long-term use of wireless telephones

Group	Total			> 5 years since first use			> 10 years since first use		
	H/L^a^	OR	95% CI	H/L^a^	OR	95% CI	H/L^a^	OR	95% CI

-Mobile telephones + DECT	56/177	1.2	0.6 – 2.4	56/170	1.2	0.6 – 2.5	40/80	1.5	0.7 – 3.1

-Men	46/58	1.5	0.6 – 3.5	46/57	1.5	0.6 – 3.6	34/39	1.6	0.6 – 3.9

-Women	10/119	0.8	0.3 – 2.6	10/113	0.8	0.3 – 2.7	6/41	1.3	0.4 – 4.6

									

-Mobile telephones	55/176	1.2	0.6 – 2.4	53/152	1.2	0.6 – 2.5	35/58	1.5	0.7 – 3.3

-Men	45/58	1.4	0.6 – 3.5	45/53	1.6	0.7 – 3.8	32/34	1.7	0.7 – 4.2

-Women	10/118	0.8	0.3 – 2.6	8/99	0.8	0.2 – 2.5	3/24	1.2	0.3 – 5.8

									

-Analogue	19/33	1.4	0.6 – 3.4	19/32	1.5	0.6 – 3.6	18/29	1.4	0.6 – 3.6

-Men	17/25	1.2	0.5 – 3.3	17/25	1.2	0.5 – 3.3	17/23	1.4	0.5 – 3.7

-Women	2/8	2.4	0.4 – 15	2/7	3.2	0.5 – 22	1/6	2.0	0.2 – 22

									

-Digital	55/173	1.2	0.6 – 2.5	53/150	1.3	0.6 – 2.6	32/52	1.6	0.7 – 3.6

-Men	45/56	1.5	0.6 – 3.6	45/52	1.6	0.7 – 3.9	29/29	1.8	0.7 – 4.6

-Women	10/117	0.8	0.3 – 2.6	8/98	0.8	0.2 – 2.5	3/23	1.3	0.3 – 6.0

									

-UMTS	13/30	1.0	0.4 – 2.9	0/0	-	-	0/0	-	-

-Men	13/17	1.6	0.5 – 5.0	0/0	-	-	0/0	-	-

-Women	0/13	-	-	0/0	-	-	0/0	-	-

									

-DECT	54/165	1.3	0.6 – 2.6	44/127	1.3	0.7 – 2.7	17/46	1.3	0.6 – 3.1

-Men	44/51	1.6	0.7 – 3.9	35/38	1.6	0.7 – 4.1	13/17	1.4	0.5 – 4.0

-Women	10/114	0.9	0.3 – 2.7	9/89	0.9	0.3 – 3.0	4/29	1.2	0.3 – 4.9

^a^H: Transthyretin > 0.31 g/L; L: Transthyretin ≤ 0.31 g/L.

Age was adjusted for in all analyses while adjustment for sex was applied only in analyses of total samples. 'Unexposed' were those with non-use and the quartile with the lowest overall use of mobile telephones and DECT (≤446 hours). Persons with missing information about use of wireless telephones were excluded from the analysis.

The linear regression analysis of cumulative hours of use gave a somewhat lower p-value for the analogue telephone type (p = 0.07) than for other telephone types (Table 4). Significantly positive β coefficients were found for time since first use of analogue telephones (p = 0.01) and for mobile telephones and DECT combined (p = 0.03). Stratification on gender gave significant findings for men and use of mobile telephones (p = 0.04), analogue telephones (p = 0.045) and mobile telephones and DECT combined (p = 0.04), but not for women. Stratification on gender for time since first use of UMTS, on the other hand, gave statistically significant associations in the opposite direction; that is, a significant negative β coefficient (p = 0.02) for men and a significant positive β coefficient (p = 0.047) for women.

**Table 4:** Linear regression analysis of serum transthyretin and long-term use of wireless telephones

Group	n	Standardized β coefficient	95% CI	p
*Cumulative use (hours), mobile telephone/DECT*				

-Mobile telephones + DECT	308	0.08	-0.02 to 0.18	0.11

-Men	132	0.10	-0.07 to 0.28	0.24

-Women	176	0.09	-0.06 to 0.23	0.25

				

-Mobile telephones	305	0.08	-0.02 to 0.19	0.11

-Men	131	0.16	-0.02 to 0.33	0.08

-Women	174	-0.02	-0.17 to 0.13	0.80

				

-Analogue	58	0.24	-0.02 to 0.50	0.07

-Men	45	0.26	-0.05 to 0.56	0.10

-Women	13	0.17	-0.53 to 0.87	0.61

				

-Digital	302	0.08	-0.02 to 0.18	0.13

-Men	130	0.15	-0.03 to 0.32	0.10

-Women	172	-0.01	-0.16 to 0.14	0.85

				

-UMTS	49	-0.11	-0.38 to 0.15	0.41

-Men	31	-0.18	-0.54 to 0.17	0.31

-Women	18	0.32	-0.29 to 0.92	0.28

				

-DECT	279	0.04	-0.07 to 0.14	0.50

-Men	117	-0.13	-0.31 to 0.06	0.17

-Women	162	0.10	-0.05 to 0.25	0.18

				

*Years since first use, mobile telephone/DECT*				

Mobile telephones + DECT	309	0.13	0.02 to 0.24	0.03

-Men	133	0.19	0.01 to 0.38	0.04

-Women	176	0.10	-0.05 to 0.25	0.20

				

-Mobile telephones	306	0.09	-0.03 to 0.20	0.13

-Men	132	0.20	0.01 to 0.38	0.04

-Women	174	0.01	-0.14 to 0.16	0.90

				

-Analogue	59	0.34	0.07 to 0.61	0.01

-Men	46	0.33	0.01 to 0.64	0.045

-Women	13	0.48	-0.18 to 1.14	0.14

				

-Digital	302	0.06	-0.05 to 0.17	0.29

-Men	130	0.14	-0.05 to 0.33	0.14

- Women	172	0.03	-0.12 to 0.17	0.74

				

-UMTS	49	-0.04	-0.30 to 0.23	0.78

-Men	31	-0.38	-0.71 to -0.06	0.02

-Women	18	0.48	0.01 to 0.94	0.047

				

-DECT	279	0.05	-0.05 to 0.16	0.33

-Men	117	0.02	-0.17 to 0.20	0.85

-Women	162	0.09	-0.07 to 0.24	0.26

Age was adjusted for in all analyses while adjustment for sex was applied only in analyses of total samples.

Data for cumulative use of mobile telephone/DECT are missing for two persons (one had used only a mobile telephone, the other had used both a mobile telephone and DECT). Data for latency of mobile telephone/DECT are missing for one person (a user of both mobile telephone and DECT).

Additional analyses with adjustments for fraction of use of the different telephone types did not change the overall results in the linear regression on cumulative hours of use and time since first use. No statistically significant result was seen for mobile telephones and DECT for cumulative use, combined (p = 0.12; data not in table), while for time since first use a significantly positive β coefficient was found (p = 0.03; data not in table). Stratification on gender gave similar findings, though for men no longer statistically significant (p = 0.08; data not in table). The results of fraction analyses showed that neither of the different types of wireless telephones altered the relative level of trend for cumulative use or time since first use, totally. However, for women a higher fraction of UMTS use raised the level both for cumulative use and time since first use (p = 0.02; 0.01 respectively; data not in table).

Further analysis yielded a statistically significant difference for serum TTR concentrations in relation to different frequencies of use in calls per day for digital (GSM) telephones (p = 0.01; Kruskal-Wallis test) but not for any of the other telephone types (data not shown). Stratification on gender gave no significant difference, however (p = 0.12 for men, p = 0.57 for women).

Participants reporting ≤ 15 min average long-term total use per day were compared with those reporting > 15 min per day (Wilcoxon rank-sum test). Here we found a statistically significant difference in TTR concentrations for digital telephones (p = 0.01, higher TTR for >15 min) and DECT (p = 0.002, lower TTR for >15 min). Again, the significances disappeared once stratification was done on gender (data not shown). However, for men, there was a tendency towards higher TTR concentrations for those who reported use of a digital telephone > 15 min per day (p = 0.06).

Logistic regression analysis of serum TTR and short-term use of wireless telephones (i.e. same day as leaving blood) yielded statistically insignificant results (Table 5). In the linear regression analysis of use of wireless telephones on the same day as giving blood, mobile telephones and DECT combined gave a low p-value with a negative β coefficient for time since most recent use (p = 0.06; Table 6), and this was significant for mobile telephone use by women (p = 0.03).

**Table 5:** Logistic regression analysis of serum transthyretin and short-term use of wireless telephones

Group	**H/L**^**a**^	OR	95% CI
Mobile telephones + DECT	55/156	1.4	0.7 – 2.8

-Men	47/55	1.8	0.8 – 4.3

-Women	8/101	0.9	0.3 – 2.8

			

-Mobile telephones	46/117	1.5	0.7 – 3.0

-Men	42/43	2.1	0.9 – 5.1

-Women	4/74	0.6	0.2 – 2.3

			

-DECT	20/79	1.2	0.5 – 2.5

-Men	15/26	1.2	0.5 – 3.3

-Women	5/53	1.0	0.3 – 3.6

^a^H: Transthyretin > 0.31 g/L; L: Transthyretin ≤ 0.31 g/L.

Age was adjusted for in all analyses while adjustment for sex was applied only in analyses of total samples.

**Table 6:** Linear regression analysis of serum transthyretin and short-term use of wireless telephones

Group	n	Standardized β coefficient	95% CI	p
*Minutes of use on the day of giving blood*				

				

Mobile telephones	163	0.01	-0.13 to 0.14	0.94

-Men	85	0.001	-0.22 to 0.22	0.99

-Women	78	0.03	-0.20 to 0.25	0.82

				

DECT	99	-0.03	-0.21 to 0.15	0.72

-Men	41	0.01	-0.32 to 0.34	0.97

-Women	58	-0.07	-0.33 to 0.19	0.59

				

*Minutes from last use – blood sampling*				

				

Mobile telephones + DECT	205	-0.11	-0.23 to 0.01	0.06

-Men	98	-0.07	-0.27 to 0.14	0.51

-Women	107	-0.21	-0.40 to -0.02	0.03

				

-Mobile telephones	159	-0.08	-0.21 to 0.06	0.25

-Men	82	0.04	-0.18 to 0.26	0.72

-Women	77	-0.25	-0.47 to -0.03	0.03

				

-DECT	97	-0.12	-0.30 to 0.06	0.20

-Men	40	-0.14	-0.47 to 0.19	0.40

-Women	57	-0.13	-0.39 to 0.13	0.33

Age was adjusted for in all analyses while adjustment for sex was applied only in analyses of total samples.

Data for minutes from last use of mobile telephone/DECT to blood sampling are missing for six persons (four had used a mobile telephone, two had used DECT).

## Discussion

There is a need to study the possible physiological effects of RF that might relate to increased risk of chronic disease in humans. Because most of the near-field RF exposure emanating from a wireless telephone is absorbed in the head of the person using the device [27], the brain is one of the main organs of interest to study. In this cross-sectional study we investigated the possible association between self-reported use of wireless telephones and serum TTR concentrations as a marker of alterations in CSF TTR, possibly reflecting an effect of RF.

Logistic regression analyses of long-term use yielded statistically insignificant results, though most ORs increased with years since first use. Linear regression analysis, on the other hand, gave significant findings for time since first use both for analogue telephones and for mobile telephones and DECT combined, which after stratification on gender only remained for men. Gender-specific analyses also gave statistically significant associations for time since first use of UMTS, but with β coefficients going in opposite directions: for men, TTR concentrations decreased significantly with increasing years, whereas for women the concentrations increased. What stands out in this group is that it consisted of significantly younger persons than the participants as a whole, see Table 1 (median age for men = 41.0 vs. 50.0; for women = 35.5 vs. 46.0). However, it is the result for men, the negative β coefficient that deviates from the general trend. Adjusting the gender-specific analysis for age, hypertension, BMI, smoking and intake of oral snuff did not change the results. Possibly the maximum time of four years since first use is too short, and that in combination with the low numbers makes the analysis sensitive to single deviant values. In women, for example, one subject with a high TTR concentration contributed strongly to the significantly positive β coefficient (included = 0.48; excluded = 0.20), and when this value was omitted the significance disappeared (p = 0.49). There was no such obvious outlier for men, so the association is either real or more likely confounded by some unknown factor possibly associated with age (median UMTS = 41 years, mobile telephones and DECT combined = 50, analogue telephones = 54).

Regarding the analysis of long-term average digital telephone use, higher TTR concentrations were found for those who reported > 15 min per day. When we stratified on gender these significances disappeared, but there was still a tendency towards higher concentrations in men. The corresponding results for use of DECT, however, showed significantly decreased concentrations of TTR in the group who reported > 15 min use per day. That significance also disappeared when men and women were analyzed separately. The result was most probably explained by the fact that the group who used DECT > 15 min per day was dominated by women (81 out of 117), who had lower TTR concentrations than men in general.

Analysis of short-term use of mobile telephone and DECT combined (min since last call) yielded a negative β coefficient, significantly so for women, indicating that the sooner blood was withdrawn after the most recent telephone call, the higher the expected TTR concentration. Since TTR has a biological half-life of 48 hours [20] it should be a reliable marker of a short-term effect. By that we mean that its rate of metabolism would not obviate the possibility of detecting an effect.

Thus, we have somewhat intriguing results for long-term use of mobile telephones (years since first use), found only in men, and short-term use (the same day as giving blood), found only in women. Regarding long-term use, the differences between men and women might (except for UMTS) be explained by the fact that men had higher TTR concentrations than women in general, which was significantly associated with time since first use. A weak short-term effect might thus be obscured.

The results might also have been influenced by confounding. The stratified analyses of the different telephone types could be a problem because of the consecutive marketing of these devices. Most users of the analogue telephone later switched to digital (GSM) and UMTS; this overlap could introduce confounding. Therefore, we also performed the additional analysis with adjustment for fractions of use of the different telephone types, which did not modify the effect for cumulative use or years since first use for mobile telephone and DECT, combined. Taking into account the fraction of each telephone did not alter the level of the overall trend either, except for women where the fraction of UMTS use raised the overall trend. However, also here, the significance disappeared when the outlier previously mentioned was omitted: for cumulative use p = 0.10; for latency p = 0.08. Thus, while the results of the separate analyses of different telephone types might be subject to confounding, this does not affect the statistically significant result for years since first use for all types, combined.

The rationale for stratifying the analyses on different generation telephones, to begin with, was that they might vary significantly in what modulation and output power they use, or used. The analogue telephone especially, uses a higher output level and lacks the adaptive power control of the more recent telephones. It also uses a lower frequency for transmission than e.g. the GSM when operating at 1800 MHz and the UMTS at 1900 MHz, which affects the specific absorption rate (SAR) – distribution in the head; the lower the frequency the deeper the penetration of radiation energy. In Sweden, the analogue system (NMT; Nordic Mobile Telephone System) was closed down in the end of 2007 after use since 1981; NMT 450 MHz 1981–2007, NMT 900 MHz 1986 – 2000. Of course, many other factors contribute to variations in the SAR-distribution of a wireless telephone, not the least how one positions the phone at the head. Also, with respect to the more recent telephone with large differences in design, probably no typical exposure exists for sub-regions of the brain, like the CP that expands throughout the ventricle system. What can be said about the exposure in these deeper parts of the brain is that it is non-thermal, and probably, in the range of 0.01–0.001 W/kg [28], but might be higher in CP of the fourth ventricle as the cerebellum is one of the brain structures that is exposed the most [27].

Intake of hormonal drugs, especially by women, was not assessed in this study and could also have introduced confounding. Estrogen use is common among the elderly and estrogens have been reported to up-regulate TTR synthesis in CP and liver in mice [29,30]. Elevated levels of TTR have also been reported in women using hormonal contraceptive drugs [31].

Nutritional status is another source of possible confounding, which was not assessed in this study. Most of the TTR in the blood is produced by the liver and is regulated independently of production by the CP. TTR from the liver is a marker of malnutrition and inflammatory processes [20]. However, our study comprised healthy population-based subjects so it is rather unlikely that malnutrition influenced the results significantly. Moreover, for hormonal contraceptives and nutritional status – or any other factor – to confound the results, those factors would have to covary with wireless telephone use (or start of use).

To avoid potential confounding by gender-associated factors, stratified analyses adjusted for age were performed throughout. Because the results were not changed, no additional adjustment was made for hypertension, BMI, smoking or use of oral snuff, even though they were statistically significantly related to TTR concentrations. Interestingly, we found higher concentrations of TTR in subjects who had ever smoked or used oral snuff. As nicotine has been reported to up-regulate the synthesis and secretion of TTR in the CP [32], these results support our choice of method using serum TTR as a marker of TTR in CSF. Moreover, a study on rats showed that nicotine did not up-regulate synthesis of TTR in the liver, only in CP [33].

Another shortcoming of our study could be that the serum TTR concentration is expected to be 10-fold higher than the CSF level. Nevertheless, the highly vascularised CP and the CSF turnover of four times per 24 hours [23], together with the 13 times faster synthetic rate of TTR in CP than in liver [34], would make alterations of TTR in the CSF detectable in the serum. If we assume that these alterations were caused by RF exposure, there are at least a couple of potential mechanisms. One is up-regulation of the TTR gene in epithelial cells; the other is dysfunction of the BCSFB leading to increased leakage or turnover of TTR in either the CP and/or the superior sagital sinuses due to the higher pressure in the subarachnoid space [17].

Ideally, TTR should be analyzed in both CSF and serum. For ethical reasons this is obviously not possible unless an opportunity is provided by patients undergoing CSF analysis for medical purposes. Another possibility would be proteomics to distinguish TTR from CP and CSF. Alternatively, use of the ratios between TTR and albumin, TTR and retinol binding protein, and TTR and thyroxin in the blood could be used to determine the origin of the TTR. We are now considering how these alternatives may be explored in further studies along with additional markers of BCSFB dysfunction.

The initial aim of the present study was to analyze serum S100B levels as a marker of BBB dysfunction and use of wireless telephones. The more general shortcomings related to weaknesses in exposure assessment, study design and low response rate have been discussed more in detail in our previous publication [4]. Briefly, use of wireless telephones was self-reported and not validated by operator records; exposure misclassification would thus be expected to some extent, although this error is unlikely to have been different with respect to serum TTR concentration since exposure was assessed beforehand. Therefore, misclassification would most likely be random, leading to a weaker association – given that there is an association. While it is a strength that the study was population-based, the low response rate of 31.4% makes the findings less valid for generalization to the whole population. Possibly it also reduces the power of the study.

## Conclusion

In conclusion, the main results of this hypothesis-generating descriptive study were that time since first use of mobile telephones and DECT combined was significantly associated with higher TTR levels regardless of how much each telephone type had been used. With regard to short-term use significantly higher TTR concentrations were seen in women the sooner blood was withdrawn after the most recent telephone call on that day.

## Abbreviations

BBB: Blood-brain barrier; BCSFB: Blood-cerebrospinal fluid barrier; BMI: Body Mass Index; CSF: Cerebrospinal fluid; CI: Confidence Interval; DECT: Digital Enhanced Cordless Telecommunications; GSM: Global system for mobile communication; MHz: megahertz; min: Minutes; NMT: Nordic Mobile Telephone System; N: Number; OR: Odds Ratio; p: Probability; RF: Radiofrequency fields; TTR: Transthyretin; UMTS: Universal Mobile Telecommunication System.

## Competing interests

The authors declare that they have no competing interests.

## Authors' contributions

FS was the principal investigator responsible for design, conduct, analysis, interpretation of data and writing the manuscript. MC participated as statistician and in the compilation and interpretation of the data and LH made contributions to conception, design, analysis of data and drafting the manuscript.
